# Peroxidase Enzyme Fractions as Markers of Somatic Embryogenesis Capacities in Olive (*Olea europaea* L.)

**DOI:** 10.3390/plants10050901

**Published:** 2021-04-29

**Authors:** Sara Oulbi, Kaoutar Kohaich, Mohammed Baaziz, Ilham Belkoura, Kenza Loutfi

**Affiliations:** 1 Laboratoire d’Agroalimentaire, Biotechnologies et Valorisation des Bio-Ressources Végétales, Faculté des Sciences Semlalia, Université Cadi Ayyad, BP 2390, Marrakech 40000, Morocco; loutfi@uca.ac.ma; 2 Laboratoire de Culture In Vitro, Département des Sciences de Base, Ecole Nationale d’Agriculture, BP S/40, Meknes 50001, Morocco; bilham@enameknes.ac.ma; 3 Laboratoire d’Amélioration Génétique des Plantes, CRRA-Marrakech, UR Amélioration des Plantes et de la Qualité, Institut National de la Recherche Agronomique, PB 533, Marrakech 40000, Morocco; 4 Laboratoire de Biochimie et Biotechnologies des Plantes, Faculté des Sciences Semlalia, Université Cadi Ayyad, BP 2390, Marrakech 40000, Morocco; kaoutar.kohaich@ced.uca.ma (K.K.); baaziz@uca.ac.ma (M.B.)

**Keywords:** *Olea europaea*, olive cultivars, somatic embryogenesis, embryogenic calluses, peroxidase, electrophoresis

## Abstract

As part of the search for biochemical markers of somatic embryogenesis in tissue cultures of olive (*Olea europaea* L.), peroxidases (POXs) in both the soluble and ionically wall-bound fractions were studied in two reputed olive cultivars (cvs.): “Picholine Marocaine” and “Dahbia”. In order to carry out embryogenesis induction, proximal cotyledons were cultured in modified olive medium (OMc) supplemented with 25 μM indole-3-butylic acid (IBA) and 2.5 μM 2-isopentenyladenine (2iP), while distal leaf fragments (somatic explants) were cultured in OMc supplemented with 4.56 µM zeatin riboside (ZR) and 10.25 µM 1-naphthaleneacetic acid (NAA). Regarding embryogenic potentials, the zygotic explants (cv. Picholine Marocaine: 43.39%; cv. Dahbia: 53.41%) were more regenerative than the somatic explants (cv. Picholine Marocaine: 13.05%; cv. Dahbia: 19.51%). The enzyme assay showed a higher POX activity in embryogenic calluses (ECs) than in nonembryogenic calluses (NECs) for the zygotic explants in both studied cultivars. When expressed as units per milligram of proteins (U mg^−1^ proteins), the highest total POXs activities (soluble POXs + ionically wall-bound POXs) were found in the ECs derived from the zygotic explants; for cv. Dahbia, 65% of the enzyme activities came from the ionically wall-bound fractions. Polyacrylamide gel electrophoresis showed that the ECs of the highly active cv. Dahbia were characterized by highly active isoperoxidases that were revealed in four migration zones, particularly a doublet in the A4 zone (R_f_ 0.70–0.73) present in the ionically wall-bound POXs. The fast-moving anodic POXs of the ionically wall-bound fractions could be adopted as an early electrophoretic test to determine the embryogenesis capacities in olive tissue culture materials. As biochemical markers, the POX enzyme and its profile in fractions, i.e., as soluble POXs and ionically wall-bound POXs, can offer a valuable tool for improving the tissue culture of olive via somatic embryogenesis.

## 1. Introduction

The olive tree (*Olea europaea* L.) is a very old culture that covers over 11.4 million hectares worldwide, where more than 96% of the global total is found in the Mediterranean basin [1]. In addition to its agronomic characteristics, the fruit and its extracted oil have a variety of interesting nutritional qualities, such as conferred by bioactive compounds, which are substances mostly known for having a large number of beneficial health properties that contribute to the protective effect of virgin olive oil [2]. Thus, it is one of the most important species grown in Morocco, even if the production levels in orchards are still remarkably low. The genetic improvement of olive tree is based on cultivar breeding programs targeting exceptional agronomic characteristics, such as to increase fruit production and the quality of extracted oil while reducing the alternation and resistance to pests and abiotic stress [3]. However, these programs are time-consuming due to the long juvenile period of the olive plant; thus, the use of in vitro culture techniques represents an alternative tool for improvement this species.

The various biotechnological tools include the use of somatic embryogenesis, which is a morphogenetic process that leads to embryo production by somatic cells and, eventually, the development of a whole plant [4,5]. This technique is considered very effective and is the basis of all biotechnological tools applied for large-scale clonal propagation, genetic transformation, somaclonal variation, in vitro selection, in vitro mutagenesis, and cryopreservation. However, the olive tree is a difficult species to handle in vitro, and the expression of somatic embryogenesis has been observed in specific explants and genotypes and under very specific growing conditions [6,7,8]. Most investigations have involved somatic embryogenesis using immature zygotic embryos [8] or radicle and cotyledon segments from mature embryos [9,10,11,12,13,14,15,16,17]. In olive plant species, somatic embryogenesis from adult tissues is a difficult process and, up to now, has only been reported in a few cases: from a double-regeneration system involving the Italian cvs. Canino and Moraiolo, by Rugini [18]; from leaves and petioles taken from the in vitro shoots of cvs. Dahbia [19], Picual [20], and Picholine Marocaine [21] and from wild olive [22,23].

In tissue culture, plants lose their juvenility, becoming more recalcitrant to de novo regeneration [24,25,26]; thus, the expression of olive somatic embryogenesis is still too low, in addition to the low germination and conversion rates of embryos to plants. Therefore, it is important to detect new biochemical markers that can orientate the choice of culture media and allow the distinction between ECs and NECs in order to profit from all of the advantages offered by the somatic embryogenesis technique. In these cases, comparative studies have been performed to distinguish ECs and NECs [27,28]. Stress response proteins have been used to identify the ECs and NECs of *Vitis vinifera* L. [29]. Zhang et al. [30] suggested that ABA induces the production of H_2_O_2_ and other active oxygen species and mediates catalases and superoxide dismutase as well as the expression of ascorbate peroxidase genes during the somatic embryogenesis of *Larix leptolepis*.

As an example of stress enzymes, POXs (POX: EC 1.11.1.7) are a class of oxidoreductases and heme-containing enzymes with the capacity to oxidize both phenols and amines with hydrogen peroxide [31]. They are involved in the regulation of many physiological processes [32], including de novo regeneration [33] and lignification [34,35]. POXs have been proposed as markers for the embryogenesis process in the tissue culture of different plant species, thus allowing the distinction between NECs and ECs [36,37]. The authors of these studies explained that POXs are involved in the regulation and differentiation of somatic embryogenic cells. However, to date, there are no reports on the study of biochemical parameters involved in the somatic embryogenesis of olive tree for improving this process.

In this study, somatic embryogenesis was induced using two types of explant taken from two Moroccan olive cultivars known for their interesting agronomic characteristics. Picholine Marocaine is the olive cultivar most cultivated in Morocco. It has a particular notoriety due to its superior qualities of fruits and oil, and it can be adapted to the environmental conditions of different regions. Dahbia, with a high production potential, is considered a good pollinator of Picholine Marocaine and is characterized by early bud break. Herein, an investigation was conducted toward understand the differences between NECs, ECs, and somatic embryos (Es) via the quantitative and qualitative analysis of POXs.

## 2. Results

### 2.1. Somatic Embryogenesis Development from Zygotic and Somatic Explants

**Figure 1:**
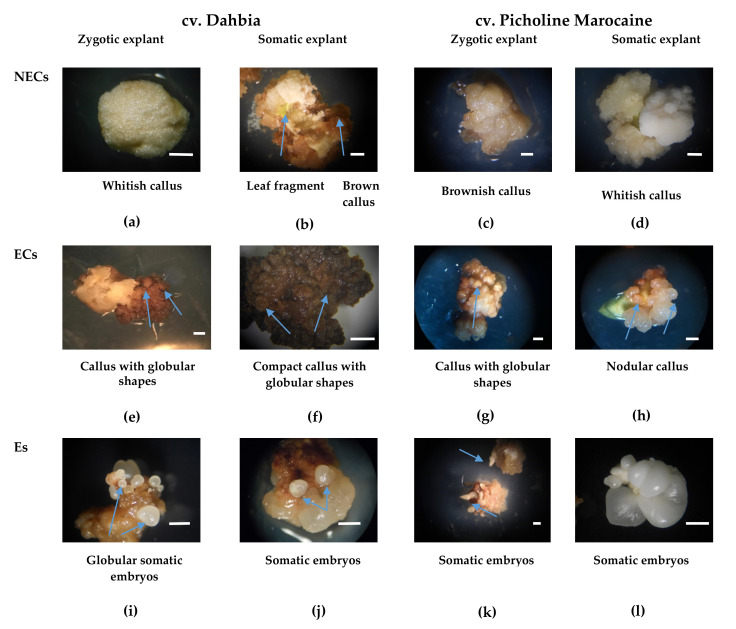
Morphological characteristics of the nonembryogenic calluses (NECs), embryogenic calluses (ECs), and somatic embryos (Es) obtained from the zygotic and somatic explants of two olive cvs. Dahbia and Picholine Marocaine, cultivated on expression medium after 4 weeks of culture in an induction medium for zygotic explants and 6 weeks for somatic explants, followed by 4 weeks of culture in a proliferation medium subculture from the induction to proliferation medium. (**a**) Spongy and white NECs obtained from zygotic explant of cv. Dahbia, (**b**) Brown NECs obtained from somatic explants of cv. Dahbia, (**c**,**d**) Friable NECs obtained from both zygotic and somatic explants of cv. Picholine Marocaine, (**e**,**f**) ECs of cv. Dahbia with abundant development of nodular structures, (**g**,**h**) Nodular structures developed on ECs of cv. Picholine Marocaine, (**i**,**j**) Globular somatic embryos observed on both, zygotic and somatic explants of cv. Dahbia, (**k**,**l**) Somatic embryos with different shapes obtained from cv. Picholine Marocaine. The bars correspond to 1 mm. The arrows on the ECs and Es indicate structures with a nodular appearance and Es, respectively.

In this study, the NECs, ECs, and Es were obtained during the somatic embryogenesis process from the distal leaf fragments and proximal cotyledons of the olive cultivars—Dahbia and Picholine Marocaine. The ECs produced Es, while the NECs exhibited no morphogenesis. After their formation, the Es were removed from the ECs. Morphological observations allowed a comparison to be made between callogenesis and somatic embryogenesis in cvs. Dahbia and Picholine Marocaine. The NECs obtained from the zygotic explants of cv. Dahbia appeared to be spongy and white (Figure 1a), while the calluses developed in the somatic explant formed brown structures (Figure 1b). However, the calluses developed from cv. Picholine Marocaine were friable and more whitish (Figure 1c,d). The ECs derived from the explants, whether somatic or zygotic, exhibited nodular structures, which were more abundant and voluminous for the cv. Dahbia (Figure 1e–h). The zygotic and somatic explants of Dahbia produced globular Es of different sizes (Figure 1i,j), which appeared in different stages of development in the case of cv. Picholine Marocaine (Figure 1k,l).

The two Moroccan olive cvs. Picholine Marocaine and Dahbia were compared based on their callogenesis and embryogenesis potentials during the in vitro tissue culture, which was characterized using zygotic and somatic explants. Globally, the Dahbia cv. was found to be more active than the Picholine Marocaine cv. (Table 1). The rates of callogenesis (induction, proliferation, and expression phases) were high for cv. Dahbia compared with cv. Picholine Marocaine when considering the zygotic and somatic explants. The rates of callogenesis in the expression phase were found to be 78.16% and 82.67% for the Picholine Marocaine and Dahbia olive cvs., respectively; when using the somatic explants in tissue culture, however, no significant difference was observed. Based on the embryogenic potential, the zygotic explants were more active than the somatic explants, with respective rates of 43.39% and 13.05% for cv. Picholine Marocaine and 53.41% and 19.51% for cv. Dahbia. The analysis of variance showed a significant effect (*p* < 0.05) of the explant nature on somatic embryo formation, and an effect of the cultivar on somatic embryogenesis was also observed. However, based on the statistical analysis, there was no apparent interaction effect for cultivar × explant. The Student–Newman–Keuls (SNK) test defined three different groups, each containing explants with the same somatic embryogenesis competence: the first regrouped the zygotic explants from cv. Dahbia, the second regrouped the zygotic explants from cv. Picholine Marocaine, and the third regrouped the somatic explants from both cultivars (Table 1).

### 2.2. Peroxidase Extraction and Enzyme Activity Test

The quantitative and qualitative aspects of the POXs extracted from the NECs, ECs, and regenerated Es were studied using the two explant types of the olive cvs. Picholine Marocaine and Dahbia. The soluble and ionically wall-bound POXs were compared.

**Figure 2:**
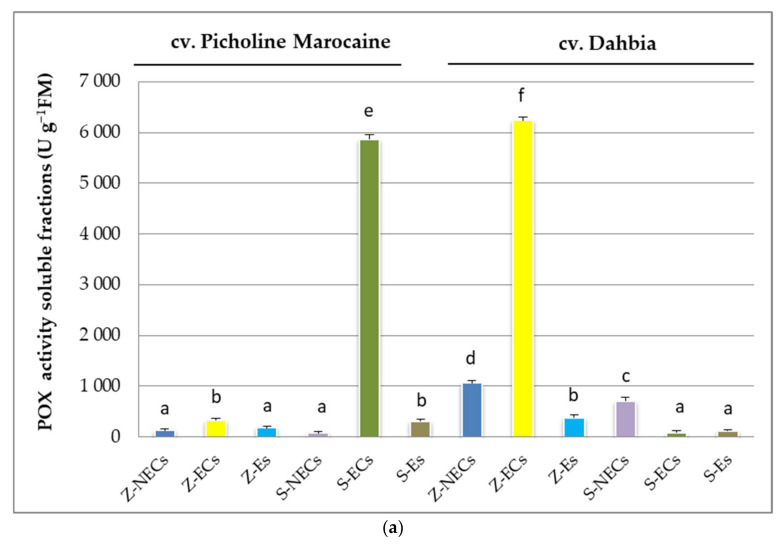

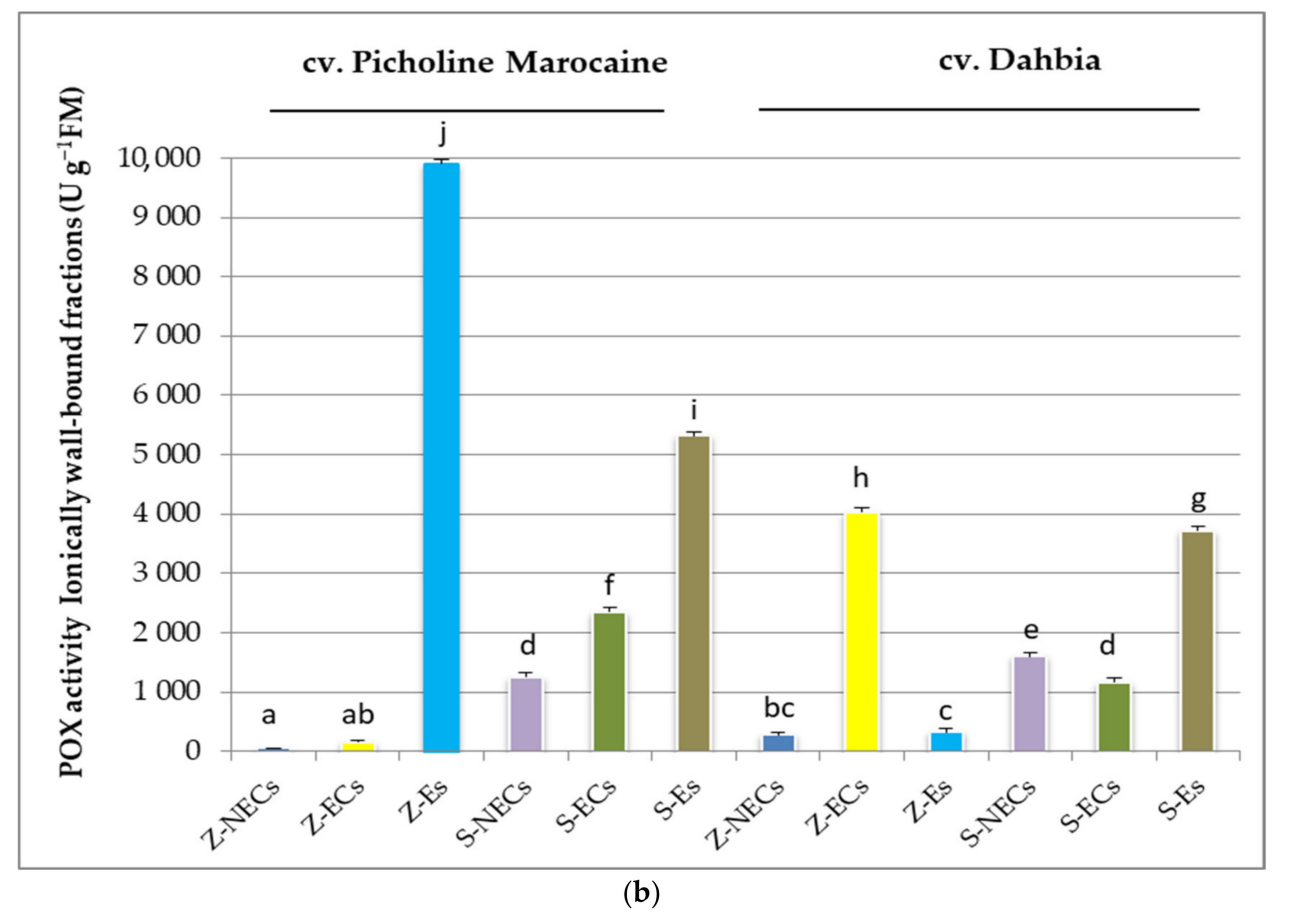
Peroxidases (POXs) activities expressed as units per gram of fresh matter (U g^−1^ FM) tested in soluble enzyme fractions (**a**) and ionically wall-bound fractions (**b**) extracted from the nonembryogenic calluses (NECs), embryogenic calluses (ECs), and somatic embryos (Es) derived from the zygotic and somatic explants of the olive cvs. Picholine Marocaine and Dahbia. Z and S refer to zygotic and somatic origins, respectively. The values refer to means of the three biological extractions. Values (mean ± SE) with different letters (a,b,c,…) are significantly different (*p* < 0.05), according to the Student–Newman–Keuls (SNK) test.

Soluble POXs were highly represented in the ECs derived from the somatic explants of Picholine Marocaine and the ECs from the zygotic explants of Dahbia (Figure 2a). In the case of the cv. Picholine Marocaine, the soluble POXs obtained from the NECs of the somatic explants were 75 times less active than those from the ECs. In the case of the cv. Dahbia, a significant difference was determined between the NECs and ECs; in particular, the NECs derived from the zygotic explants were 6 times less enzymatically active than the ECs of the same explants. Regenerated Es were typified by low levels of soluble POXs. However, an inverse tendency was encountered with the ionically wall-bound POXs, where Es showed high levels of enzyme activities followed by the ECs derived from the somatic explants of the Dahbia and Picholine Marocaine cvs. (Figure 2b). Thus, the ECs derived from the zygotic explants of the cv. Dahbia showed significantly higher POX activities than those of the NECs, and they registered activities more than 14 times higher than those found in the NECs.

**Figure 3:**
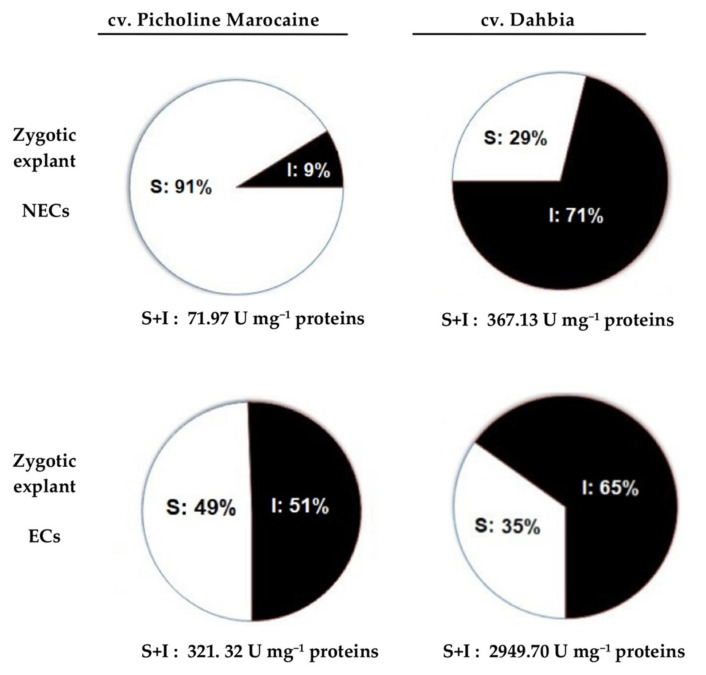
Percentages of peroxidases (POXs) activities (U mg^−1^ proteins) tested in soluble (S) and ionically wall-bound (I) enzyme fractions extracted from the nonembryogenic calluses (NECs) and embryogenic calluses (ECs) derived from the zygotic explants of the olive cvs. Picholine Marocaine and Dahbia.

The POXs tested in the NECs, ECs, and Es showed significantly different activities according to the olive cultivars and explant types used in the tissue culture (Table 2). Generally, ECs and Es were often richest in soluble and ionically wall-bound POXs. The total POX activities (S+I) of the ECs derived from the zygotic explants represented more than 4.5 and 8 times the activities of the NECs found in cvs. Picholine Marocaine and Dahbia, respectively. The total POX activity of the ECs derived from the somatic explant was 2.6 times higher than that found in the NECs for cv. Picholine Marocaine. When compared with the ECs derived from the zygotic explants of cv. Picholine Marocaine, the same calluses obtained from cv. Dahbia were typified by significantly high POX activities for both the soluble and ionically wall-bound enzyme fractions. This tendency was different in the somatic explants. Each in vitro tissue material could be characterized by the percentage (%) of soluble and ionically wall-bound fractions from the total POX activities (S+I). For example, the Es obtained from the somatic explant of the cv. Picholine Marocaine were characterized by low levels of soluble POXs (85 U mg*^−^*^1^ proteins) and significantly high levels of ionically wall-bound POXs (1531.03 U mg*^−^*^1^ proteins) (Table 2). When compared to the NECs obtained from the zygotic explants of the cv. Dahbia, the ECs derived from the same explant were typified by 65–71% of the ionically wall-bound POXs fractions and only 29–35% of the soluble POXs fractions (Figure 3). The analysis of variance at a significance level of 5% showed a significant effect (*p* < 0.05) of the cultivar and the explant nature on the POX activity fractions. The olive cv. Dahbia with a relatively high embryogenic potential was marked by high contents of total POX. Contrarily to the zygotic explants, the somatic explants (with low embryogenesis capacities) produced unbalanced parts of soluble and ionically wall-bound POXs in the ECs (85.36% soluble and 14.64% ionically wall-bound for cv. Picholine Marocaine; 7.81% soluble and 92.19% ionically wall-bound for cv. Dahbia) (Table 2).

### 2.3. Electrophoresis of Peroxidases

**Figure 4:**
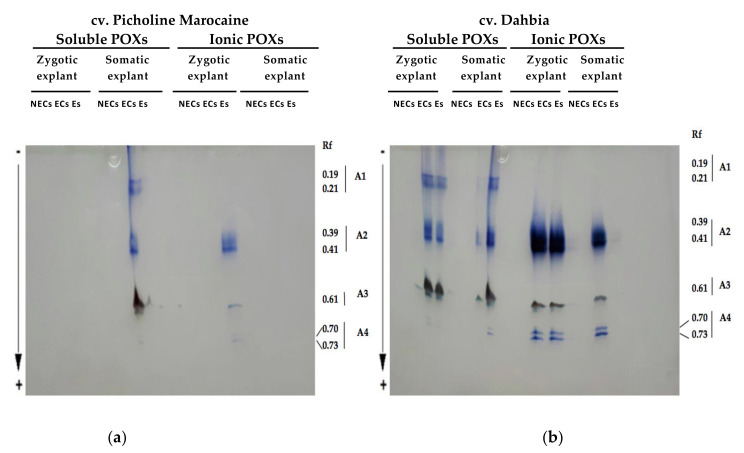
Zymograms of the soluble and ionically wall-bound peroxidases (POXs) of the olive cvs. Picholine Marocaine (**a**) and Dahbia (**b**) obtained by electrophoresis on polyacrylamide gels. Enzyme fractions were extracted from the nonembryogenic calluses (NECs), embryogenic calluses (ECs), and somatic embryos (Es) derived from the zygotic and somatic explants of each cultivar.

To test the involvement of POXs in olive somatic embryogenesis, the soluble and ionically wall-bound POX fractions were investigated during this process. Thus, native gel electrophoresis was used to resolve the different isoperoxidases of the acidic soluble POX and the ionically wall-bound POX, typifying calluses and Es. At first glance, the polyacrylamide gels reflected a quantitative aspect, considering the in situ-revealed band intensities, where the olive cv. Dahbia showed high POX activities compared to the cv. Picholine Marocaine (Figure 4). The POX zymograms exhibited four zones of activity named A1 (R_f_ 0.19–0.21), A2 (R_f_ 0.39–0.41), A3 (R_f_ 0.61), and A4 (R_f_ 0.70–0.73), showing acidic POXs that migrate toward the anode on basic gels. The gels of the cv. Picholine Marocaine, which has a relatively low embryogenesis capacity, contained few isoperoxidases, limited to ECs and Es (Figure 4a). However, the gels of the cv. Dahbia, a cultivar with relatively high embryogenesis capacities, were rich in isoperoxidases (Figure 4b).

When compared with soluble POX, ionically wall-bound POXs were typified by the presence of two active isoforms in the A4 zone and the absence of isoperoxidases in the A1 zone. The isoenzyme profiles of the calluses and Es derived from the zygotic and somatic explants were qualitatively similar. Electrophoresis showed that the ECs were characterized by highly active isoperoxidases revealed in four migration zones for the cv. Dahbia (high embryogenesis capacities), particularly a doublet in the A4 zone (R_f_ 0.70–0.73) presented in the ionically wall-bound enzyme fractions. The electrophoresis experiment showed that these enzyme fractions are suitable for determining the embryogenesis capacities in olive trees.

## 3. Discussion

The main objective of this work was to investigate POXs enzymes as putative biochemical markers of somatic embryogenesis, regarding quantitative and qualitative aspects, by investigating their expression in two types of explants of two Moroccan olive cultivars. When zygotic explants were used, the results showed that the embryogenesis capacity was significantly different between the two cultivars, confirming the difference in the embryogenic competence according to juvenile plant material of the cultivars. According to multiple authors, somatic embryogenesis depends on the genotype and, therefore, on the cultivar [14,38,39,40]. However, it has been reported that many olive cultivars are recalcitrant to in vitro culture and, especially, to the induction of somatic embryogenesis, while others respond perfectly to this regeneration process and with a higher embryogenic potential [14,35,39]. For many plants, genotype-dependent somatic embryogenesis is related to the accumulation of some transcripts such as *CmAGL11* (affecting the expression of several genes related to the gibberellin, auxin, and ethylene pathways) [41] or to chemical compounds and proteins, including antioxidants [42,43]. Olive embryogenic competence is also determined by the juvenility of the used explant, where differences between cultivars have not been noted in somatic embryo initiation when using immature zygotic embryos [8]. However, a significant effect of the cultivar has been observed when conducting somatic embryogenesis based on mature zygotic embryos [15]. Herein, based on morphological observations, the embryogenic cultures expressed nodular calluses in the expression phase for ECs, and with increasing culture time, these structures became more brownish and exhibited nodular structures that invaded the entire explant. Sánchez-Romero [44] reported the development of Es, individually for necrotic calluses and nodular structures, after transferring to expression conditions for several weeks. The NECs did not show a notable proliferation, but the ECs showed competence in the formation of new proembryogenic masses or secondary Es, as was reported by Sánchez-Romero [44].

In this study, cv. Dahbia showed a greater embryogenic competence than cv. Picholine Marocaine when zygotic explants were used, indicating that, under the same culture conditions, the capacity varies according to the genotype, as reported by Bradaï et al. [45,46], and the type of explant [47]. The genotype influenced the developmental stages and number of Es. The callogenesis capacity did not show significant differences between the two varieties, and the formation rate of calluses remained higher throughout the embryogenesis process and in all used explants. In fact, cv. Dahbia produced globular Es of different sizes, while the somatic embryogenesis was asynchronous in the case of the cv. Picholine Marocaine, when globular, heart, and torpedo Es coexisted on the same calluses, as was reported for olive by different authors [10,13,14,44].

Somatic embryogenesis is relatively possible from juvenile explants; however, obtaining embryogenic cultures is difficult when the starting material is mature tissue. In recent years, a few studies have been carried out on somatic embryogenesis from mature tissues collected from wild [22] and cultivated olive [19,20].

The results show that the expression of Es also depends on the nature of the explants (proximal cotyledons and distal leaf fragments). The embryogenic competence of calluses and embryo formation were higher in the case of juvenile plant material using zygotic Es for the induction of embryogenesis. This could be due to exogenous growth regulators, which modify the concentrations of endogenous hormones that depend on the nature of the explants to form Es [48]. The addition of plant growth regulators to the expression medium is important, as reported in several studies [17,49].

Some studies on somatic embryogenesis indicate that there are some morphological and cellular criteria that characterize the acquisition of embryogenic competence for different plants. Namasivayam [50] reported that there is no universal physiological or structural marker that can clearly indicate a suitable cell candidate for embryo formation. The morphological characteristics of calluses can provide information about their embryogenic competence. However, this is not sufficient to make a constructive conclusion. Thus, biochemical or molecular markers have become necessary to demonstrate the embryogenic potential and to offer a tool to carry out follow-ups of the embryogenic capacity [51,52,53,54].

In this study, POXs enzymes, oxidoreductases enzymes involved in plant growth and development [55], were investigated as possible valuable biochemical markers that can be used to identify the embryogenesis capacities of different calluses at an early stage. Spectrophotometric-based tests of enzyme activities expressed as units per gram of fresh matter (U g^−1^ FM) U g^−1^ FM or units per milligram of proteins U mg^−1^ proteins showed that soluble and ionically wall-bound POXs were strongly represented in the ECs and Es derived from the olive cvs. Picholine Marocaine and Dahbia. In the case of other plant species, such as *Cucurbita pepo*, the embryogenic lines showed a POX activity approximately 20 times higher than that of the nonembryogenic line [56]. In *Pinus koraiensis*, the activities of POXs and catalase trended upward during early somatic development [57]. Almeida et al. [58], studying *Medicago truncatula*, also found a high accumulation of POX in the stages coinciding with embryo formation in the embryogenic line. Therefore, the authors suggested that this higher expression level may be associated with cell wall elongation. The increase in activity in the ECs could be due to the fact that POXs have an important role during the formation of embryo cell walls [59]. In a proteomic analysis of the ECs and NECs in maize, Varhaníková et al. [60] suggested that increased POX activity correlated with the acquisition of regeneration capacity. However, there are opposite examples, where the reduction in POX activity typified ECs. Thus, Zdravković-Korać et al. [61] observed a reduction in the activity of POX (extraction with liquid nitrogen and pyrogallol as a substrate) during the transition of friable calluses to ECs of *Aesculus flava*. A similar decrease in POX activity (ascorbate peroxidase) was observed during the induction of regeneration in *Avena nuda* [62]. In addition to the plant species factor, these differences could be related to the POX type, preparation protocol, and the substrates used in activity tests. Indeed, it has been shown that the use of certain substrates in the POXs tests could produce latencies, which result in an underestimation of POXs activities [63].

The results show that the ECs derived from the zygotic explants of cvs. Picholine Marocaine and Dahbia were typified by 65–70% of the ionically wall-bound POXs fractions for both cultivars and high levels of total POXs (soluble POXs + ionically wall-bound POXs). When compared to the NECs obtained from the zygotic explants of the cv. Dahbia, the ECs derived from the same explant were typified by 51–65% of the ionically wall-bound POXs fractions and only 35–49% of the soluble POXs fractions. These results underline the importance of ionically wall-bound POXs in the embryogenesis process of olive trees. Ionically wall-bound POXs are involved in lignification as evidenced by the close correlation between lignin content and enzyme activity observed in *Vigna angularis* [59]. The higher POX activity associated with the cell wall could be associated with maintenance of the cell wall rigidity of Es. Similar results have been obtained in *Medicago arborea* L. [53], where use of the corresponding isoforms of POXs and ascorbate peroxidases as markers for the identification of embryological potential has been demonstrated. Moreover, the present results are in accordance with the positive correlation between POX activity and somatic embryogenesis in date palm [36,37].

In this study, the results for the spectrophotometry-based quantitation of POXs were confirmed using qualitative electrophoresis-based analysis of olive POXs. Highly active isoperoxidases were derived from the cv. Dahbia (a cultivar with high embryogenesis capacities) for ECs, particularly a doublet in the A4 zone and in the highly intense A2 zone in the ionically wall-bound POXs. This latter enzyme fraction is most interesting compared with the soluble enzyme fraction. In other studies, new POXs isoforms have been found in regenerating explants [64].

## 4. Materials and Methods

### 4.1. Plant Material and Culture Establishment

#### 4.1.1. Somatic Embryogenesis from Zygotic Material

The plant material used during the experiments was collected from young fruits of the olive cvs. Picholine Marocaine and Dahbia. The fruits were harvested during the first week of October 2017 from healthy adult trees from the Agro-Pôle Olivier ENA-Meknès collection. Olive fruits were harvested by hand, where good-quality fruits were chosen and placed in plastic bags to be transported directly to the in vitro culture laboratory. Before their use, the fresh mature fruits were thoroughly washed under running tap water and then disinfected as follows: The fruits were removed from their pulp, and the olive pits were kept for 12 h in commercial bleach (38 g L^−1^ sodium hypochlorite) and then crushed to extract the seeds, which were subsequently sterilized with commercial bleach at a concentration of 10% for 10 min. These were then rinsed three times with sterile distilled water for 10 min. The extracted olive seeds were soaked in sterile distilled water for 48 h to promote their swelling and to facilitate the extraction of zygotic embryos. Before the excision of the embryos (zygotic explant), a second sterilization was carried out by commercial bleach at a concentration of 10% for 10 min, followed by three baths of sterile distilled water.

The preparation of the plant material was carried out under laminar flow. A binocular magnifier and sterile dissection equipment were used to isolate the zygotic embryos and dissect them into three distinct parts: radicle, proximal, and distal cotyledons. The radicle and proximal parts of the cotyledons were cultured. The regeneration rate of radicles was low (data not shown); therefore, only proximal cotyledons (zygotic explants) were processed in this study. Modified olive medium (OMc) [65] was used for the induction of somatic embryogenesis, supplemented with the following hormonal combinations: induction medium (IM): OMc + 25 μM IBA + 2.5 μM 2 iP [40]. After 4 weeks of culture, the calluses obtained on these media were subcultured on the OMc medium supplemented with 2.5 μM of IBA for 4 weeks; 30 g L^−1^ of sucrose was used as a carbon source and the media were solidified with 6 g L^−1^ of Bactoagar. Then, the cultures were transferred to olive cyclic embryogenesis medium (ECO) as the expression medium (EM) [66], which contained the macronutrients of OMe medium [65], 1/4 MS microelements [67], 1/2 OM vitamins, 50 mg L^−1^ of myo-inositol, 550 mg L^−1^ of glutamine, and 1 g L^−1^ of casein hydrolysate, supplemented with 0.25 μM of IBA, 0.5 μM of 2iP, and 0.44 μM of BAP, and solidified by 3 g L^−1^ of Phytagel [47].

#### 4.1.2. Somatic Embryogenesis from Adult Tissues

The Moroccan cvs. Picholine Marocaine and Dahbia were used as mother plants: rejuvenated plantlets were obtained from cuttings and maintained for eight years under aseptic conditions. The cuttings were collected from adult and healthy tree cvs. of Dahbia and Picholine Marocaine, and were defoliated and sterilized as follows. They were soaked in 70% ethanol for 1 min and 2% calcium hypochlorite for 3 min, and were then washed three times for 10 min with sterile distilled water. To induce the development of axillary shoots, cuttings were divided into single-node microcuttings and cultured in OM supplemented with 3% sucrose and 2 mg L^−1^ zeatin riboside (ZR) [68]. To obtain rejuvenated plant material, the developed shoots were divided and subcultured for an interval of 4 weeks. The leaves were taken from these plantlets obtained from the development of the axillary buds and maintained under in vitro conditions. The leaves of the plantlets were cut into four parts: petioles, proximal leaf blades, intermediate leaf blades, and distal parts. In the results, only the distal part of the leaves (somatic explants) was used. The regeneration rate of the other parts of the leaves was low (data not shown). The explants were cultured for 6 weeks in the induction media composed of OMc supplemented with 4.56 µM ZR and 10.25 µM NAA [21], followed by the transfer to OMc supplemented with 2.5 μM IBA for 4 weeks [65]. Furthermore, 30 g L^−1^ sucrose was used as a carbon source and media were solidified with 6 g L^−1^ Bactoagar. The cultures were then transferred to the ECO expression medium with additional transfer every 4 weeks [66], supplemented with 0.25 μM IBA, 0.5 μM 2iP, and 0.44 μM BAP, and were solidified using 3 g L^−1^ Phytagel.

The calluses obtained after the induction and proliferation phases were transferred into fresh expression medium of the same type every 4 weeks. However, the formed Es were transferred into maturation medium consisting of OM supplemented with 1 g activated charcoal. In addition, 30 g L^−1^ sucrose was used as a carbon source and the medium was solidified using 6 g L^−1^ Bactoagar. The calluses and Es obtained after 4 weeks of culture on the expression medium were subjected to the extraction of POX, followed by quantitative and qualitative enzymatic tests.

Before autoclaving (121 °C for 20 min), the pH of all the media was adjusted with KOH or HCL to 5.7. All of the used reagents were purchased from Sigma-Aldrich (Steinheim, Germany). All cultures were maintained under darkness at 25 ± 1 °C.

For the two types of explants and both cvs. Dahbia and Picholine Marocaine, ECs were defined as those producing Es after four weeks of culture on the expression medium. However, NECs were those that did not allow the development of Es after the same period, while Es were extracted from the surface.

### 4.2. Peroxidase Extraction

The calluses obtained were used for the extraction of POXs, as described by Baaziz et al. [36]. Three biological replicates were used; each extraction was carried out based on an average of 20 calluses (NECs and ECs) or Es. Pieces of 0.25 g were ground in a cold mortar in 1.5 mL of 5 mM of Tris–HCl buffer (pH 7.2) containing 0.25 M of sucrose and 1 mM of MgCl_2_. A plant weight-to-volume ratio of 1:6 was used in the enzyme extraction. The supernatant collected after centrifugation for 7 min at 9000× *g* corresponds to the soluble enzyme fraction. After washing the pellet three times in 1.5 mL of 5 mM of Tris–HCl buffer (pH 7.2) containing 1% (*v*/*v*) of Triton X-100, the residue was incubated for 30 min in 1.5 mL of 5 mM of Tris–HCl (pH 7.2) and 1 M of NaCl. The supernatant obtained after centrifugation according to the above corresponds to the ionically wall-bound POXs fractions [69].

### 4.3. Enzyme Activity Test of Peroxidases

The POX activity was carried out spectrophotometrically using guaiacol as the substrate [63]. The variation in absorbance allowed the POXs activities to be calculated as units per gram of fresh matter (U g^−1^ FM) or units per milligram of proteins (U mg^−1^ proteins). One unit of POX activity was defined as the quantity of enzyme that catalyzes a change in absorbance at 470 nm equal to 0.01 min^−1^. Tests were performed using biological triplicates.

Protein determination was achieved according to the Lowry method [68] by measuring the optical density at 750 nm with the bovine serum albumin as standard.

### 4.4. Electrophoresis and Staining Gels for Peroxidase Isoenzymes

The qualitative aspect of POX was achieved by electrophoresis. Acidic isoperoxidases were separated by electrophoresis (Pharmacia system supporting two gels) at +4 °C, using 6%–15% gradient polyacrylamide gels prepared in 0.37 M of Tris–HCl buffer (pH 8.8), as described by Baaziz et al. [36] and Majourhat et al. [69]. Samples were loaded on gels by volume (50 μL). In the final step of electrophoresis, the gels were stained for POXs isoforms using 0.05% benzidine and 1% hydrogen peroxide as substrates [63,69]. The in situ enzyme activity was photographed after 15 min of incubation.

### 4.5. Statistical Analysis

A completely randomized design was used for the experiment. The enzyme data were determined in triplicate. The somatic embryogenesis experiments consisted of 20 replicates comprising 20 Petri dishes containing 5 explants each and repeated thrice for each type of explant (zygotic and somatic explants) and each cultivar. A total of 600 explants were used. The biochemical data and callogenesis in the induction, proliferation, and expression phases, in addition to the E percentage, were calculated and compared to the total number of initially cultured explants, and they were subjected to analysis of variance (ANOVA). Prior to analysis, the percentage data were arcsine-transformed. Means were separated by the Student–Newman–Keuls (SNK) test at the 5% significance level. All statistical calculations were made using SPSS (v. 26, IBM, Chicago, IL, USA).

## 5. Conclusions

This study presents the first report of the involvement of POXs—oxidoreductases enzymes—in the process of somatic embryogenesis in olive tree, previously induced from two types of explants belonging to two important Moroccan olive cultivars. The obtained results showed that the ECs and Es of the olive cultivars were typified by high levels of total POXs, both soluble and ionically wall-bound fractions, compared with the NEC ones. Considering the results obtained in the present study, POXs, particularly ionically wall-bound POXs, could serve as a biochemical marker for the acquisition of *Olea europaea* L. olive tissues capable of undergoing somatic embryogenesis. Thus, we propose that an early electrophoresis test of the ionically wall-bound POXs could be used to assess olive embryogenic capacities and save time in the propagation of this species through somatic embryogenesis. This finding can offer a new perspective and a significant advance in the optimization of the expression of somatic embryogenesis by different tissues of *Olea europaea* L., and it can contribute to the enhancement of large propagations of olive genetic resources through tissue cultures.

## Figures and Tables

**Table 1:** Rates (%) of callogenesis and embryogenesis obtained for the zygotic (proximal cotyledons) and somatic (distal leaf fragments) explants of the olive cvs. Dahbia and Picholine Marocaine, on the induction (4 weeks for the zygotic explants and 6 weeks for the somatic explants), proliferation (4 weeks for both types of explants), and expression (4 weeks with refreshment for both types of explants) phases of the culture.

Cultivar	Explant Type	Callogenesis			Embryogenesis
		Induction	Proliferation	Expression	Somatic Embryos Formation
Picholine Marocaine	Zygotic explants	79.77 ± 0.24	80.72 ± 0.26	81.15 ± 0.18	43.39 ± 0.14 ^b^
	somatic explants	75.17 ± 0.20	77.13 ± 0.23	78.16 ± 0.18	13.05 ± 0.24 ^c^
Dahbia	Zygotic explants	80.09 ± 0.25	81.26 ± 0.27	84.89 ± 0.21	53.41 ± 0.23 ^a^
	Somatic explants	77.84 ± 0.26	79.36 ± 0.21	82.67 ± 0.13	19.51 ± 0.19 ^c^

Statistical analysis of data was carried out after arcsine transformation. Values (mean ± SE) with different letters (a,b,c,…) are significantly different (*p* < 0.05) according to the Student–Newman–Keuls (SNK) test.

**Table 2:** Protein concentrations (mg mL^−1^) and peroxidases (POXs) activities (U mg^−1^ proteins) tested in soluble and ionically wall-bound enzyme fractions extracted from the nonembryogenic calluses (NECs), embryogenic calluses (ECs), and somatic embryos (Es) derived from the zygotic and somatic explants of the olive cvs. Picholine Marocaine and Dahbia.

			cv. Picholine Marocaine		cv. Dahbia	
			Protein (mg mL^−1^)	POXs Activities (U mg^−1^ Proteins) *	Protein (mg mL^−1^)	POXs Activities (U mg^−1^ Proteins) *
Soluble enzyme fractions (S)	Zygotic explant	NECs	0.32 ± 0.02 ^e^	65.63 ± 6.36 ^c^ (91.19%)	1.66 ± 0.11 ^e^	106.02 ± 12.73 ^c^ (28.88%)
		ECs	0.34 ± 0.03 ^b^	158.82 ± 8.49 ^e^ (49.43%)	1.01 ± 0.22 ^b^	1029.70 ± 16.97 ^e^ (34.91%)
		Es	0.12 ± 0.07 ^a^	241.67 ± 10.61 ^d^ (2.43%)	0.8 ± 0.09 ^a^	78.75 ± 14.85 ^d^ (50.06%)
	Somatic explant	NECs	0.66 ± 0.06 ^d^	19.70 ± 6.36 ^b^ (3.18%)	1.05 ± 0.24 ^d^	112.38 ± 16.97 ^b^ (16.81%)
		ECs	0.71 ± 0.11 ^c^	1377.46 ± 21.21 ^f^ (85.36%)	0.75 ± 0.02 ^c^	18.67 ± 8.49 ^f^ (7.81%)
		Es	0.60 ± 0.05 ^f^	85.00 ± 10.61 ^a^ (5.26%)	1.54 ± 0.37 ^f^	12.99 ± 4.24 ^a^ (1.60%)
Ionically wall-bound enzyme fractions (I)	Zygotic explant	NECs	1.42 ± 0.44 ^e^	6.34 ± 2.12 ^a^ (8.81%)	0.18 ± 0.01 ^e^	261.11 ± 10.61 ^a^ (71.12%)
		ECs	0.16 ± 0.03 ^a^	162.50 ± 8.49 ^d^ (50.57%)	0.35 ± 0.07 ^a^	1920.00 ± 16.97 ^d^ (65.10%)
		Es	0.17 ± 0.05 ^c^	9711.76 ± 19.09 ^f^ (97.57%)	0.7 ± 0.05 ^c^	78.57 ± 14.85 ^f^ (49.94%)
	Somatic explant	NECs	0.35 ± 0.02 ^b^	600.00 ± 16.97 ^c^ (96.82%)	0.48 ± 0.01 ^b^	556.25 ± 14.85 ^c^ (83.18%)
		ECs	1.66 ± 0.48 ^f^	236.14 ± 21.21 ^b^ (14.64%)	0.88 ± 0.21 ^f^	220.45 ± 21.21 ^b^ (92.19%)
		Es	0.58 ± 0.12 ^d^	1531.03 ± 12.73 ^e^ (94.74%)	0.78 ± 0.12 ^d^	796.15 ± 14.85 ^e^ (98.40%)

* Values in parentheses refer to the percentages of each enzyme fraction from the total POXs. Values (mean ± SE) refer to the means of three biological extractions. Values (mean ± SE) with different letters (a,b,c,…) are significantly different (*p* < 0.05) according to the Student–Newman–Keuls (SNK) test.

## Data Availability

Not applicable.
